# The Forgotten Role of Alcohol: A Systematic Review and Meta-Analysis of the Clinical Efficacy and Perceived Role of Chlorhexidine in Skin Antisepsis

**DOI:** 10.1371/journal.pone.0044277

**Published:** 2012-09-05

**Authors:** Matthias Maiwald, Edwin S. Y. Chan

**Affiliations:** 1 Department of Pathology and Laboratory Medicine, KK Women’s and Children’s Hospital, Singapore, Singapore; 2 Department of Microbiology, National University of Singapore, Singapore, Singapore; 3 Department of Epidemiology, Singapore Clinical Research Institute, Singapore, Singapore; 4 Singapore Branch, Australasian Cochrane Centre, Singapore, Singapore; 5 Duke-National University of Singapore Graduate Medical School, Singapore, Singapore; Aligarh Muslim University, India

## Abstract

**Background:**

Skin antisepsis is a simple and effective measure to prevent infections. The efficacy of chlorhexidine is actively discussed in the literature on skin antisepsis. However, study outcomes due to chlorhexidine-alcohol combinations are often attributed to chlorhexidine alone. Thus, we sought to review the efficacy of chlorhexidine for skin antisepsis and the extent of a possible misinterpretation of evidence.

**Methods:**

We performed a systematic literature review of clinical trials and systematic reviews investigating chlorhexidine compounds for blood culture collection, vascular catheter insertion and surgical skin preparation. We searched PubMed, CINAHL, the Cochrane Library, the Agency for Healthcare Research and Quality website, several clinical trials registries and a manufacturer website. We extracted data on study design, antiseptic composition, and the following outcomes: blood culture contamination, catheter colonisation, catheter-related bloodstream infection and surgical site infection. We conducted meta-analyses of the clinical efficacy of chlorhexidine compounds and reviewed the appropriateness of the authors′ attribution.

**Results:**

In all three application areas and for all outcomes, we found good evidence favouring chlorhexidine-alcohol over aqueous competitors, but not over competitors combined with alcohols. For blood cultures and surgery, we found no evidence supporting chlorhexidine alone. For catheters, we found evidence in support of chlorhexidine alone for preventing catheter colonisation, but not for preventing bloodstream infection. A range of 29 to 43% of articles attributed outcomes solely to chlorhexidine when the combination with alcohol was in fact used. Articles with ambiguous attribution were common (8–35%). Unsubstantiated recommendations for chlorhexidine alone instead of chlorhexidine-alcohol were identified in several practice recommendations and evidence-based guidelines.

**Conclusions:**

Perceived efficacy of chlorhexidine is often in fact based on evidence for the efficacy of the chlorhexidine-alcohol combination. The role of alcohol has frequently been overlooked in evidence assessments. This has broader implications for knowledge translation as well as potential implications for patient safety.

## Introduction

Skin antisepsis has been an indispensable part of medical practice for more than a century. After a period of increased attention in the 1970s and 1980s that temporarily waned, there is now renewed interest in its role as a simple and effective measure for preventing healthcare-acquired infections.

The most commonly used substances for skin antisepsis are (1) alcohols (ethanol, isopropanol and n-propanol), (2) chlorhexidine, commonly available as chlorhexidine gluconate (CHG), and (3) povidone-iodine (PVI), an organic iodine complex. Among these antiseptics, alcohols are microbiologically most active but have no appreciable residual activity [1]–[3]. CHG and PVI are less effective, but have residual activity on skin, which is pronounced for CHG but small for PVI. The usual active concentrations are about 70–90% (v/v) for alcohols, 0.5–4% (w/v) for CHG, and 5–10% (w/v) for PVI (or, instead of total PVI, 0.5–1% “available” iodine). Both CHG and PVI are available as aqueous solutions where they are the sole active ingredients, and they can be combined with alcohols, thereby creating enhanced antiseptics with two active components. There is also iodine tincture, which is an alcoholic solution of elemental iodine and potassium iodide.

Among the antiseptics, CHG has attracted considerable attention through several prominent clinical studies concerning vascular catheters and surgery [4]–[6]. CHG became a topic of discussion and a subject of keynote presentations at conferences. Preference for CHG, in particular over its main competitor, PVI, was expressed in several practice recommendations and evidence-based guidelines for skin antisepsis [7]–[10].

We noticed an inconsistent interpretation of findings in some primary studies and subsequent reviews. Several articles that evaluated the efficacy of the combination of alcohols plus CHG attributed the study outcomes solely to the CHG component [11]–[13]. These articles effectively concluded that CHG was the only agent responsible for positive outcomes and that CHG *per se* was superior to PVI *per se* when in fact CHG-alcohol versus PVI alone had been tested.

This apparent misinterpretation of evidence and an increasing number of recommendations that were focussing prominently or exclusively on the efficacy of the CHG component prompted us to reassess the evidence by way of systematic review. We posed the following questions: (1) What is the evidence for the efficacy of CHG alone or combination antiseptics containing it for blood culture collection, vascular catheter insertion, and surgical skin preparation? (2) How common is the attribution of efficacy from a combination of antiseptics to CHG alone in the primary literature and in systematic reviews? (3) Has this misattribution had an effect on practice recommendations and evidence-based guidelines?

## Methods

### Literature Search Strategy

**Figure 1:**
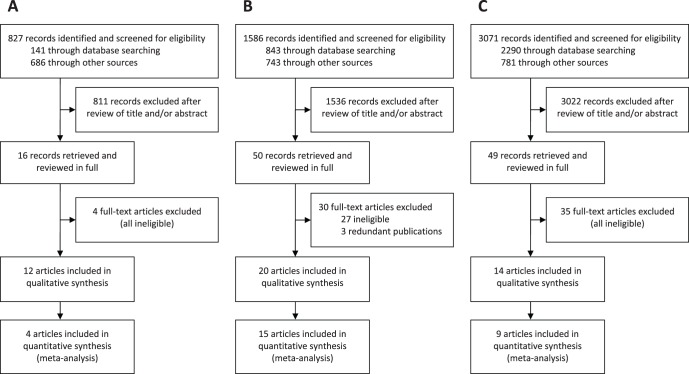
Flow diagrams of literature search and study selection in three areas of skin antisepsis. (A) blood culture collection; (B) vascular catheter insertion; (C) surgical skin preparation. Reasons for exclusion at the full-text article stage are provided in Text S1.

Exhaustive searches for primary and secondary literature were performed in three areas of skin antisepsis: (1) blood culture collection, (2) vascular catheter insertion, and (3) surgical skin preparation. For the purpose of this review, primary literature was defined as randomised clinical trials (RCTs) and non-randomised clinical studies, and secondary literature was defined as systematic reviews. Searches were performed using PubMed, CINAHL, the Cochrane Library, the Agency for Healthcare Research and Quality website, several clinical trials registries, and a CHG product manufacturer’s website (CareFusion, San Diego, CA, USA). Apart from the selection of databases, no specific limits on publication dates and language were applied. The full literature search strategy is provided in Text S1, and a PRISMA flow diagram in Figure 1. A PRISMA Checklist is provided in Checklist S1.

### Selection Criteria

All included primary and secondary articles had to have evaluated any CHG-containing antiseptic against any other antiseptic in one of the three areas of interest. The following outcomes had to be reported: (1) for blood culture studies, the rate of blood culture contamination, (2) for vascular catheter studies, the rates of microbial catheter colonisation and/or catheter-related bloodstream infection (CR-BSI), and (3) for surgery articles, the rate of surgical site infections. The following interventions were excluded: antiseptic cloth wiping or bathing in the preoperative phase, antisepsis only during catheter maintenance but not at insertion, non-superficial skin antisepsis, and where skin antisepsis was only part of a multifactorial intervention. Further information on eligibility criteria is provided in Text S1.

### Data Extraction and Quality Assessment

Data were extracted on study design, antiseptics compared and their composition, main outcomes, and the authors’ interpretation of the study results. All primary (RCTs and non-RCTs) and secondary articles were rated to assess the authors’ attribution of study outcomes from CHG-containing antiseptics (qualitative synthesis), while only RCTs were selected for subsequent meta-analyses (quantitative synthesis). All RCTs were appraised for risk of bias using a domain-based approach recommended by the Cochrane Collaboration [14]. Further details and the results of risk of bias assessment are provided in Text S1 and Tables S1, S2 and S3.

### Assessment of Authors’ Attribution (Qualitative Synthesis)

Attribution was rated as “correct” if study authors recognised that the combination of both CHG and alcohol was used and therefore responsible for the outcomes. It was rated as “incorrect” if authors clearly attributed study outcomes derived from the combination of CHG and alcohol to CHG alone. It was rated as “intermediate” if there were ambiguous statements, such as when authors recognised the antiseptic properties of alcohols but also made statements suggesting that CHG alone might be responsible. It was rated as “not applicable” if CHG alone without alcohol had been used.

### Meta-analyses of Clinical Efficacy (Quantitative Synthesis)

Meta-analyses to quantify the clinical efficacy of CHG compounds were performed using the RevMan software [15] by computing relative risks (RR) and 95% confidence intervals (CI). Only RCTs that were clinically homogenous and had tested the same basic antiseptic components were pooled together. In the absence of statistical heterogeneity, a fixed-effects model was used for analysis, and in the presence of statistical heterogeneity (I^2^≥50%; p≤0.1), both fixed-effects and random-effects models were used in a sensitivity analysis.

### Survey of Tertiary Literature

The impact of the conclusions in the primary and secondary literature on perceptions in the medical community and on practice recommendations was gleaned from a non-exhaustive survey of the tertiary literature. Tertiary literature was defined as any other articles commenting on the role of CHG in skin antisepsis, including narrative reviews, professional websites and e-mail discussion forums, clinical practice recommendations, and evidence-based guidelines.

## Results

### Skin Antisepsis for Blood Culture Collection

A total of 12 articles met the inclusion criteria for blood culture collection; this included 10 primary studies [11], [16]–[24] and two systematic reviews [25], [26] (Table 1). Among the primary studies, four were RCTs. All of the articles evaluated CHG-alcohol combinations, none evaluated aqueous CHG.

**Table 1:** Primary studies and systematic reviews evaluating chlorhexidine-containing antiseptics for the prevention of blood culture contamination.

Reference^a^	Study design	Antiseptics compared^b^	Main outcomes^c^	Comments	Attribution^d^
Mimoz et al. 1999 [16] (M, C)	RCT	A: CHG 0.5% + ALC (?%); B: PVI aq 10%	A: 14/1019; B: 34/1022; p<0.05	Advantage of CHG + ALC over PVI aq	Incorrect
Trautner et al. 2002 [17] (M, C)	RCT^e^	A: CHG 2% + IPA 70%; B: IPA 70% seq IT (I_2_ 2%, ETH 47%)	A: 1/215; B: 3/215; NS	Study design equivalent to RCT	Correct
Barenfanger et al. 2004 [18]	Non-RCT^f^	A: CHG 2% + IPA 70%; B: IT (composition?)	A: 158/5802; B: 186/5936; NS	Composition of IT could not be clarified	Incorrect
Madeo et al. 2008 [19]	Non-RCT^f^	A: CHG 2% + IPA 70%; B: Unknown	A: 40/1870; B: 304/4072; p<0.05	Weak study design, comparator unknown	Correct
McLellan et al. 2008 [20]	Non-RCT^f^	A: CHG 2% + IPA 70%; B: IPA 70%	Complex outcomes^g^	Weak study design, thoughtful analysis	Correct
Stonecypher 2008 [21]	Non-RCT^f^	A: CHG 2% + IPA 70%; B: PVI aq 10%	A: 23/687; B: 37/612; p<0.05	Alcohol in arm A only revealed by correspondence	Incorrect
Suwanpimolkul et al. 2008 [22] (C)	RCT	A: CHG 0.5% + ETH 70%; B: PVI aq 10%	A: 34/1068; B: 74/1078; p<0.05	Advantage of CHG + ALC over PVI aq	Correct
Tepus et al. 2008 [23]	Non-RCT^f^	A: CHG 2% + IPA 70%; B: IPA 70% seq IT (I_2_ 2%, ETH 47%)	A: 169/7606; B: 251/7158; p<0.05	Confounder: staff training before CHG + IPA study arm	Intermediate
Marlowe et al. 2010 [11]	Non-RCT^f^	A: CHG 3.15% + IPA 70%; B: PVI aq 10%	A: 72/4274; B: 122/4942; p<0.05	Attribution criticised in letter to the editor	Incorrect
Washer et al. 2010 [24]	Cluster-randomised cross-over trial	A: CHG 2% + IPA 70%; B: IPA 70% seq PVI aq 10% ; C: IPA 70% seq IT (I_2_ 2%, ETH 50%)	A: 41/4347; B: 25/4261; C: 32/4198; all NS	Use of IPA before PVI and IT in arms B and C, clarified by author	Correct
Malani et al. 2007 [25]	Systematic review	4 eligible trials, 2 with CHG-containing antiseptics	No clear evidence; possible benefits from packaged kits and alcohol-based antiseptics	Results overall inconclusive	Correct
Caldeira et al. 2011 [26]	Systematic review	6 eligible trials, 3 with CHG-containing antiseptics	Alcoholic products > non-alcoholic ones; ALC + CHG > PVI aq; CHG compounds vs iodine compounds inconclusive; ALC alone not inferior to iodine products	Article appropriately analyses different ingredients and compositions of antiseptics	Correct

ALC, alcohol (when alcohol type not known); aq, aqueous; CHG, chlorhexidine gluconate; ETH, ethanol; IPA, isopropanol; IT, iodine tincture; PVI, povidone iodine; RCT, randomised clinical trial; seq, sequential application; vs, versus; ?%, percentage not specified; > (in systematic reviews), performing better than.

a Annotation with (M) or (C) denotes whether original studies were included in the systematic reviews of Malani et al [25] (M) or Caldeira et al [26] (C).

b A, B, and C denote different study arms.

c Outcome: number of contaminated blood cultures per cultures obtained in each study arm. Significance is indicated either by NS (not significant) or p<0.05 (when significant).

d Attribution: assesses whether study outcomes derived from alcohol plus CHG were attributed to CHG alone by authors.

e In this trial, all subjects received both antiseptics at the same time, outcomes were assessed blindly.

f These studies were classified as non-randomised cluster cross-over trials. Some had been conducted by prospective sequential implementation of different antiseptic regimens in clinical units [18], [20], [21], some by retrospective comparison of antiseptic regimens [11], [19], [23].

g This study had complex outcomes from several pre- and post-intervention intervals showing that rigorous training and application may be more important than the choice of antiseptic.

Correct attribution was found in seven articles (58%), ambiguous statements (intermediate ranking) in one (8%), and incorrect attribution in four (33%). Among the ones with incorrect attribution, three noted the presence of alcohol in the CHG-containing preparation but did not associate it with the efficacy of the antiseptic, while one published abstract listed and discussed CHG alone, and the presence of alcohol was found out through correspondence. Both systematic reviews recognised the importance of alcohols.

**Figure 2:**
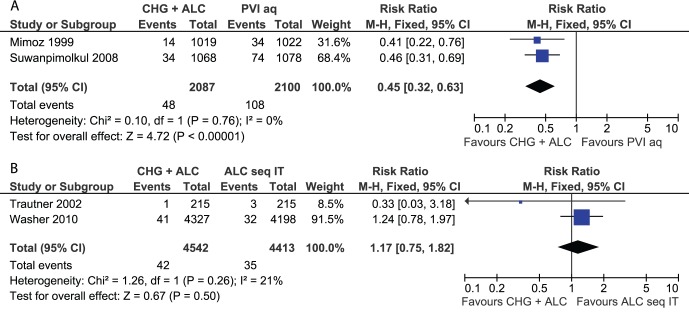
Meta-analyses of skin antiseptics for the prevention of blood culture contamination. (A) CHG plus alcohol versus aqueous PVI. (B) CHG plus alcohol versus sequential alcohol followed by iodine tincture. References and abbreviations are as provided in Table 1.

Two parallel-group RCTs 16,22, one within-subject trial with each subject experiencing both interventions [17] and a cluster-randomised cross-over trial [24] were subjected to meta-analyses (Figure 2). The results showed that the combination of CHG plus alcohol was significantly better than aqueous PVI alone (RR: 0.45; 95% CI: 0.32–0.63) and that there was no significant difference between CHG-alcohol versus sequential isopropanol and iodine tincture (RR: 1.17; 95% CI: 0.75–1.82). A single comparison of CHG-alcohol versus sequential isopropanol and PVI [24] also showed no significant difference (RR: 1.61; 95% CI: 0.98–2.64). The results of the non-RCTs are listed in Table 1 but were not included in meta-analyses.

The Malani et al systematic review [25] included four trials, two examining CHG-containing antiseptics. The authors found no clear evidence favouring any particular type of antiseptic, however, they identified possible benefits from prepackaged kits and alcohol-containing antiseptics. The Caldeira et al review [26] included six trials, three examining CHG-containing antiseptics. Several conclusions were made: (1) alcoholic iodine tincture was better than aqueous PVI, (2) alcoholic CHG was better than aqueous PVI, (3) alcoholic products were better than non-alcoholic ones, and (4) alcohol alone was not inferior to any iodine products. The authors commented that alcohol alone may be sufficient.

We identified several tertiary sources that contained unsubstantiated statements concerning the efficacy of CHG. A Clinical and Laboratory Standards Institute (CLSI) guideline for blood culture collection [7] stated: “chlorhexidine gluconate [without reference to the presence of alcohol]... is the recommended skin disinfectant for older infants, children, and adults”. A standard textbook on phlebotomy [27] contained similar statements. Several contributions to the discussion forum ClinMicroNet (American Society for Microbiology) discussed “chlorhexidine” (without reference to alcohols) and its benefits for blood culture collection.

In contrast, we did not find any relevant evidence supporting the use of CHG alone prior to blood culture collection.

### Skin Antisepsis for Vascular Catheter Insertion

A total of 20 articles met the inclusion criteria for vascular catheter insertion; this included 18 primary studies [28]–[45] and two systematic reviews [46], [47] (Table 2). Among the primary studies, 15 were RCTs. Four studies evaluated aqueous CHG, 13 evaluated CHG-alcohol combinations, and two evaluated a triple combination of CHG, benzalkonium chloride and benzyl alcohol. There were four studies with three study arms.

**Table 2:** Primary studies and systematic reviews evaluating chlorhexidine-containing antiseptics for the prevention of intravascular catheter-associated infections.

Reference^a^	Study design^b^	Antiseptics compared^c^	Outcomes catheter colonisation^d^	Outcomes CR-BSI^d^	Comments	Attribution^e^
Maki et al. 1991 [28] (C)	RCT; CVCs, ACs; insertion and maintenance	A: CHG aq 2%; B: PVI aq 10%; C: IPA 70%	A: 5/214; B: 21/227; C: 11/227; only A:B p<0.05	A: 1/214; B: 6/227; C: 3/227; all NS	Seminal study; only arms A vs B in colonisation significant	Not applicable
Sheehan et al. 1993 [29] (C)	RCT; CVCs, ACs; insertion and maintenance	A: CHG aq 2%; B: PVI aq 10%	A: 3/169; B: 12/177; p<0.05	A: 1/169; B: 1/177; NS	Conference abstract; colonisation significant	Not applicable
Garland et al. 1995 [30]	Non-RCT; PVCs; only insertion, not maintenance^f^	A: CHG 2% + IPA 70%; B: PVI aq 10%	A: 20/418; B: 38/408; p<0.05	A: 2/418; B: 0/408; NS	Only colonisation significant	Incorrect
Meffre et al. 1996 [31] (C)	RCT; PVCs; insertion and maintenance	A: CHG 0.5% + ALC (?%); B: PVI aq 10%	A: 9/568; B: 22/549; p<0.05	A: 3/568; B: 3/549; NS	Conference abstract; colonisation significant	Correct
Mimoz et al. 1996 [32] (C)	RCT; CVCs, ACs; insertion and maintenance	A: CHG 0.25% + BAK 0.025% + BALC 4%; B: PVI aq 10%	A: 12/170; B: 24/145; p<0.05	A: 3/170; B: 3/145; NS	Synergistic combination of three antiseptics in arm A	Correct
Legras et al. 1997 [33] (C)	RCT; CVCs, ACs; insertion and maintenance	A: CHG 0.5% + ALC (?%); B: PVI aq 10%	A: 19/179; B: 31/224; NS	A: 0/208; B: 4/249; NS	Differences non-significant	Intermediate
Cobbett and LeBlanc 2000 [34] (C)	RCT; PVCs; insertion yes, maintenance not specified	A: CHG 0.5% + IPA 70%; B: ALC (?%) seq PVI aq 10%; C: PVI aq 10% seq ALC (?%)	A: 6/83; B: 12/80; C: 11/81; All NS	ND	Differences non-significant, also when B and C pooled vs A	Correct
Humar et al. 2000 [35] (C)	RCT; CVCs; insertion and maintenance	A: CHG 0.5% + ALC (?%); B: PVI aq 10%	A: 36/116; B: 27/116; NS	A: 4/193; B: 5/181; NS	Differences non-significant; sole study with slight disadvantage of CHG + ALC vs PVI aq	Intermediate
Maki et al. 2001 [36] (C)	RCT; CVCs, PICCs, ACs; insertion and maintenance	A: CHG 1% + ALC 75%; B: PVI aq 10%	A: 43/422; B: 192/617; p<0.05	A: 4/422; B: 23/617; p<0.05	Largest study; biggest difference between study arms	Intermediate
Langgartner et al. 2004 [37] (R)	RCT; CVCs; insertion was studied; maintenance all with CHG + ALC	A: CHG 0.5% + IPA 70%; B: PVI aq 10%; C: CHG 0.5% + IPA 70% seq PVI aq 10%	A: 11/45; B: 16/52; C: 2/43; A:C, B:C p<0.05	ND	Arm C (sequential protocol) significantly better than A or B	Correct
Astle and Jensen 2005 [38] (R)	RCT; CVCs (hemodialysis); insertion and maintenance	A: CHG 0.5% + IPA 70%; B: ExSept	ND	A: 1/64; B: 1/57; NS	Study did not report catheter colonisation	Incorrect
Kelly et al. 2005 [39]	RCT; CVCs, ACs; insertion and maintenance	A: CHG 2% + IPA 70%; B: PVI aq 10%	A: 4/82; B: 15/82; p<0.05	A: 1/82; B: 8/82; p<0.05	Conference abstract; alcohol in arm A only revealed by correspondence	Incorrect
Balamongkhon et al. 2007 [40]	Non-RCT; insertion and maintenance^f^	A: CHG 2% + ETH 70%; B: PVI aq 10%	ND	A: 3/120; B: 7/192; NS	Weak study design, difference non-significant	Intermediate
Mimoz et al. 2007 [41] (R)	RCT; CVCs; insertion and maintenance	A: CHG 0.25% + BAK 0.025% + BALC 4%; B: PVI 5% + ETH 70%	A: 28/242; B: 53/239; p<0.05	A: 4/242; B: 10/239; NS	Rare study with PVI-alcohol; difference for colonisation significant	Intermediate
Small et al. 2008 [42] (R)	RCT; PVCs; only insertion, not maintenance	A: CHG 2% + IPA 70%; B: IPA 70%	A: 18/91; B: 39/79; p<0.05	ND	Significant difference; but mean colony counts lower in IPA alone group	Correct
Vallés et al. 2008 [43] (R)	RCT; CVCs, ACs; insertion and maintenance	A: CHG 2% + ALC (?%); B: CHG 2% aq; C: PVI aq 10%	A: 34/226; B: 38/211; C: 48/194; only A:C p<0.05	A: 9/226; B: 9/211; C: 9/194; all NS	Only difference in arms A vs C in colonisation significant	Correct
Garland et al. 2009 [44]	RCT; PICCs; insertion and maintenance	A: CHG 0.5% + ALC (?%); B: PVI aq 10%	A: 3/24; B: 1/24; NS	A: 0/24; B: 0/24; NS	Small study; focus on skin tolerability in neonates	Incorrect
Ishizuka et al. 2009 [45]	Non-RCT; CVCs; insertion studied; maintenance all PVI aq^f^	A: CHG aq 0.05%; B: PVI aq 10%	ND	A: 14/286; B: 6/298; NS	CHG concentration very unusually low	Not applicable
Chaiyakunapruk et al. 2002 [46]	Systematic review	8 eligible trials, 2 with CHG aq, 1 with CHG plus other compounds, 5 with CHG + ALC; comparator for all PVI aq 10%	Relative risk for CHG-containing vs PVI aq was about 0.5 (50%) for colonisation and CR-BSI	See comments under colonisation	Seminal review; basis for multiple recommendations; only CHG + ALC but not CHG aq significant in CR-BSI	Incorrect
Rickard and Ray-Barruel 2010 [47]	Systematic review	7 eligible trials, 5 examined any CHG-containing antiseptic prior to catheter insertion	Any CHG vs any others performed significantly better in colonisation but not in CR-BSI; same for any CHG vs any PVI	See comments under colonisation	Article available on internet; part of Australian national infection control guidelines	Intermediate

ACs, arterial catheters; ALC, alcohol (when alcohol type not known); aq, aqueous; BAK, benzalkonium chloride; BALC, benzyl alcohol; CHG, chlorhexidine gluconate; CR-BSI, catheter-related bloodstream infection; CVCs, central venous catheters; ETH, ethanol; IPA, isopropanol; ND, not determined; PICCs, peripherally inserted central venous catheters; PVCs, peripheral venous catheters; PVI, povidone iodine; RCT, randomised clinical trial; seq, sequential application; vs, versus; ?%, percentage not specified.

a Annotation with (C) or (R) denotes whether original studies were included in the systematic reviews of Chaiyakunapruk et al [46] (C) or Rickard and Ray-Barruel [47] (R).

b Mention of insertion and maintenance refers to whether the assigned study antiseptic was used prior to catheter insertion only, or both, for insertion and maintenance.

c A, B, and C denote different study arms.

d Outcome: number of catheters colonised or CR-BSIs per catheters inserted in each study arm. Significance is indicated either by NS (not significant) or p<0.05 (when significant).

e Attribution: assesses whether study outcomes derived from alcohol plus CHG were attributed to CHG alone by authors.

f These studies were classified as non-randomised cluster cross-over trials. They had been conducted by prospective sequential implementation of different antiseptic regimens in clinical units.

Judgement of attribution was not applicable to three studies, as they used aqueous CHG only. Among the remaining 17 articles, correct attribution was found in six articles (35%), ambiguous statements (intermediate ranking) in another six articles (35%), and incorrect attribution in five articles (29%). Three original articles correctly listed the presence of alcohol but did not associate it with antiseptic efficacy, while for one abstract, the presence of alcohol was found out through correspondence.

**Figure 3:**
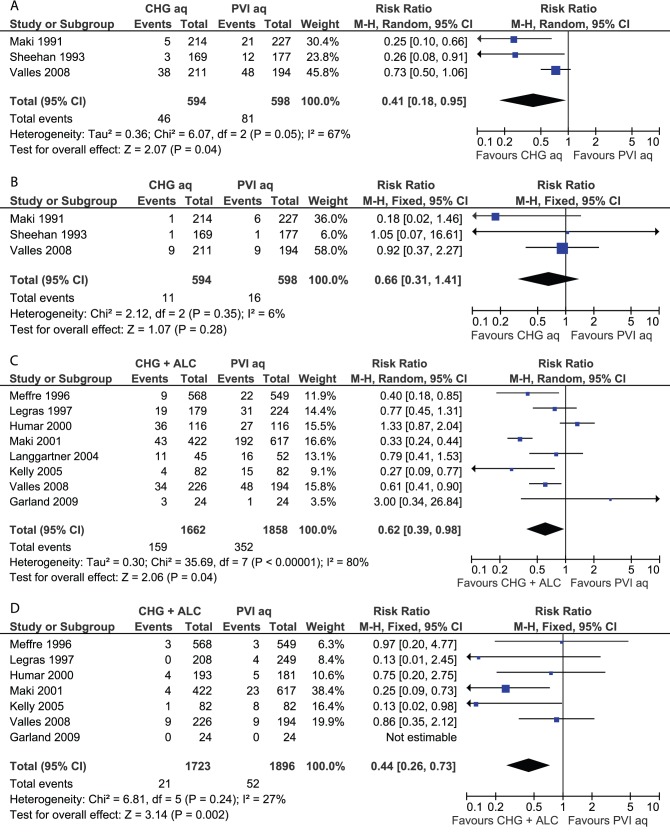
Meta-analyses of skin antiseptics for the prevention of vascular catheter-related infection. (A) Aqueous CHG versus aqueous PVI, outcome catheter colonisation. (B) Aqueous CHG versus aqueous PVI, outcome catheter-related bloodstream infection. (C) CHG plus alcohol versus aqueous PVI, outcome catheter colonisation. (D) CHG plus alcohol versus aqueous PVI, outcome catheter-related bloodstream infection. References and abbreviations are as provided in Table 2.

Four meta-analyses were performed (Figure 3); this included two analyses (catheter colonisation and CR-BSI) on aqueous CHG versus aqueous PVI (3 trials each) and two analyses on CHG-alcohol versus aqueous PVI (7 and 8 trials, respectively). The comparison of aqueous CHG with aqueous PVI indicated a significantly lower risk of catheter colonisation in the CHG group (RR 0.41; 95% CI: 0.18–0.95), but lacked significance for CR-BSI (RR 0.66; 95% CI: 0.31–1.41). The comparison of CHG-alcohol with PVI alone indicated significant benefits for CHG plus alcohol for both catheter colonisation (RR 0.62; 95% CI: 0.39–0.98) and CR-BSI (RR 0.44; 95% CI: 0.26–0.73). Statistical heterogeneity was detected in both groupings for the outcome of catheter colonisation. For aqueous CHG versus aqueous PVI (Figure 3A), the source of heterogeneity appeared to be the trial of Vallés et al [43], for CHG-alcohol versus aqueous PVI (Figure 3C), potential sources were the trials of Humar et al [35] and Maki et al [36].

Additional single-trial comparisons included (1) the combination of CHG, benzalkonium chloride and benzyl alcohol versus aqueous PVI [32], showing a benefit for the CHG preparation for catheter colonisation (RR 0.43; 95% CI: 0.22–0.82) but not for CR-BSI (RR 0.85; 95% CI: 0.17–4.16), (2) the same combination versus PVI plus alcohol [41], showing a benefit for the CHG preparation for colonisation (RR 0.52; 95% CI: 0.34–0.80) but not CR-BSI (RR 0.40; 95% CI: 0.13–1.24), and (3) a trial of CHG-alcohol versus sequential alcohol and aqueous PVI [34] being insignificant for colonisation (RR 0.51; 95% CI: 0.21–1.19). Again, the results of the non-RCTs are listed individually in Table 2 but were not included in meta-analyses.

The first systematic review [46] included eight trials, two examining 2% aqueous CHG, one a triple combination with CHG, and five examining CHG-alcohol combinations. The comparator for all was 10% aqueous PVI. The authors pooled all studies and found a significant risk reduction for colonisation and CR-BSI in the CHG-containing group. However, all study outcomes were solely attributed to CHG. Only a brief passage in the Discussion mentioned that only the subset of studies testing alcoholic CHG had produced a significant reduction in CR-BSI, but not the ones testing aqueous CHG. It was concluded that this may have been due to inadequate statistical power from the smaller number of studies with aqueous CHG. The second systematic review [47] included seven trials, five examining CHG-containing antiseptics against different competitors. It compared both aqueous CHG and CHG-alcohol combinations versus different antiseptics in a non-CHG group. It found a benefit of CHG-containing solutions for preventing device colonisation.

Again, we found several examples in the tertiary literature that referred to CHG alone where the CHG-alcohol combination would have been relevant. A follow-up article [48] on the 2002 systematic review of Chaiyakunapruk et al [48] examined the clinical and economic benefits of CHG for vascular catheter site care. It commented on the benefits of CHG in preventing CR-BSI, even though that had only been demonstrated for the CHG-alcohol combination. A seminal article on the Keystone Project [4] that described evidence-based procedures to decrease CR-BSIs in 108 intensive care units mentioned skin preparation with “chlorhexidine” without referring to alcohol. In fact, almost all units had used a CHG-alcohol combination from one company (CareFusion, correspondence). Both the 2002 Centers for Disease Control (CDC) guidelines for intravascular catheters [8] and the draft of the 2011 guidelines [9] recommended preparing the skin with a 2% chlorhexidine-based preparation for central venous catheters. This was classified as Category IA evidence. The draft was followed by a public comment phase, and the final 2011 guideline [49] recommended a >0.5% chlorhexidine skin preparation with alcohol. The final guideline also stated that the relative efficacy of CHG-alcohol versus PVI-alcohol was unresolved.

Overall, we found strong evidence supporting the efficacy of CHG-alcohol antisepsis for catheter insertion and maintenance, particularly when compared with aqueous PVI. We also found evidence favouring aqueous CHG over aqueous PVI, but this was limited to the outcome catheter colonisation. In single trials, a CHG-containing triple combination was better than PVI-alcohol [41], and CHG-alcohol was better than alcohol alone [42], both in terms of catheter colonisation.

### Skin Antisepsis before Surgery

A total of 14 articles met the inclusion criteria for surgical skin antisepsis; this included 11 primary studies [6], [50]–[59] and three systematic reviews [12], [13], [60] (Table 3). Among the primary studies, 9 were RCTs. All primary articles evaluated CHG-alcohol combinations, none aqueous CHG.

**Table 3:** Primary studies and systematic reviews evaluating chlorhexidine-containing antiseptics for the prevention of surgical site infections.

Reference^a^	Study design	Antiseptics compared^b^	Main outcomes^c^	Comments	Attribution^d^
Berry et al. 1982 [50] (E, L, N)	RCT; mixed surgery, including abdominal	A: CHG 0.5% + ALC (?%); B: PVI 10% + ALC (?%)	A: 44/453; B: 61/413; p<0.05	ALC type and content in both study arms unknown; difference significant	Incorrect
Brown et al. 1984 [51] (L, N)	RCT; mixed surgery, including obstetric, abdominal	A: CHG 0.5% + IPA 70%; B: PVI aq (0.7% av I_2_) seq PVI aq (?%)	A: 23/378; B: 29/359; NS	Difference non-significant	Incorrect
Ostrander et al. 2005 [52] (L)	RCT; clean foot and ankle surgery	A: CHG 2% + IPA 70%; B: IPOV (0.7% av I_2_) + IPA 74%; C: Chloroxylenol 3%	A: 1/40; B: 0/40; C: 2/40; all NS	Also skin microbial counts studied, but methodology not adequately described	Intermediate
Veiga et al. 2008 [53] (L)	RCT; elective clean plastic surgery	A: CHG 0.5% + ALC (?%); B: PVI 10% + ALC (?%)	A: 0/125; B: 4/125; NS	Difference non-significant; ALC type and content unknown	Incorrect
Cheng et al. 2009 [54]	RCT; clean forefoot surgery	A: CHG 2% + IPA 70%; B: PVI 10% + IPA 23%	A: 0/25; B: 0/25; NS	Small study; focus on skin counts; IPA content in arm B far below active range	Intermediate
Paocharoen et al. 2009 [55] (L, N)	RCT; general surgery, including clean, clean-contaminated and contaminated cases	A: CHG 4% + IPA 70%; B: PVI aq (?%)	A: 5/250; B: 8/250; NS	Difference non-significant	Incorrect
Saltzman et al. 2009 [56] (L)	RCT; clean shoulder surgery, including arthroscopic	A: CHG 2% + IPA 70%; B: IPOV (0.7% av I_2_) + IPA 74%; C: PVI aq scrub & paint (0.75% & 1.0% av I_2_)	A: 0/50; B: 0/50; C: 0/50; NS	Small study; focus on skin counts; microbiological methods potentially inadequate	Correct
Swenson et al. 2009 [57] (N)	Non-RCT; mixed general surgery^e^	A: CHG 2% + IPA 70%; B: PVI aq 7.5% seq IPA 70% seq PVI aq 10%; C: IPOV (0.7% av I_2_) + IPA 74%	A: 68/827; B: 72/1514; C: 38/794; A:B, A:C p<0.05	Significantly more infections in CHG + ALC arm, but only superficial ones	Correct
Darouiche et al. 2010 [6] (L, N)	RCT; mixed clean-contaminated surgery, including abdominal	A: CHG 2% + IPA 70%; B: PVI aq 10% scrub & paint	A: 39/409; B: 71/440; p<0.05	Seminal study; significant difference in favour of CHG + ALC over PVI aq	Correct
Sistla et al. 2010 [58]	RCT; elective clean inguinal hernia surgery	A: CHG 2.5% + ETH 70%; B: PVI aq 10%	A: 14/200; B: 19/200; NS	Difference non-significant	Correct
Levin et al. 2011 [59]	Non-RCT; elective gynaecological laparotomy surgery^e^	A: CHG aq 2% seq IPA 70%; B: PVI aq 10% seq PVI 10% + ETH 65%	A: 5/111; B: 21/145; p<0.05	Weak study design; significant difference	Correct
Edwards et al. 2004 [60]	Systematic review	7 eligible trials, only 1 with a CHG-containing arm [50]	Overall inconclusive due to lack of well-designed studies	Review from 2004, updated 2009; lack of studies at the time	Intermediate
Lee et al. 2010 [12]	Systematic review	9 eligible trials, 5 studied CHG + ALC vs PVI aq, 4 studied CHG + ALC vs PVI + ALC (including 1 both), 1 studied CHG aq vs PVI aq for mucous membranes	“Chlorhexidine” superior to iodine, based on majority CHG + ALC vs PVI aq outcomes	Analysed both infection rates and microbial skin counts; criticised in letters to the editor	Incorrect
Noorani et al. 2010 [13]	Systematic review	6 eligible trials, 3 studied CHG + ALC vs PVI aq, 2 CHG + ALC vs PVI + ALC, 1 CHG aq vs PVI aq for mucous membranes	“Chlorhexidine” superior to iodine, based on majority CHG + ALC vs PVI aq outcomes	Attribution criticised in letters to the editor	Incorrect

ALC, alcohol (when alcohol type not known); aq, aqueous; av, available (referring to available iodine as opposed to total iodine complex); CHG, chlorhexidine gluconate; ETH, ethanol; IPA, isopropanol; IPOV, iodine povacrylex; PVI, povidone iodine; RCT, randomised clinical trial; seq, sequential application; vs, versus; ?%, percentage not specified.

a Annotation with (E), (L), or (C) denotes whether original studies were included in the systematic reviews of Edwards et al [60] (E), Lee et al [12] (L), or Noorani et al [13] (N).

b A, B, and C denote different study arms.

c Outcome: surgical site infections per number of surgical procedures in each study arm. Significance is indicated either by NS (not significant) or p<0.05 (when significant).

d Attribution: assesses whether study outcomes derived from alcohol plus CHG were attributed to CHG alone by authors.

e These studies were classified as non-randomised cluster cross-over trials. One had been conducted by prospective sequential implementation of different antiseptic regimens in clinical units [57] and one by retrospective comparison of antiseptic regimens after sequential implementation [59].

Among all primary and secondary articles, correct attribution to both CHG and alcohol was found in five articles (36%), ambiguous statements (intermediate ranking) in three articles (21%), and incorrect attribution in six articles (43%).

**Figure 4:**
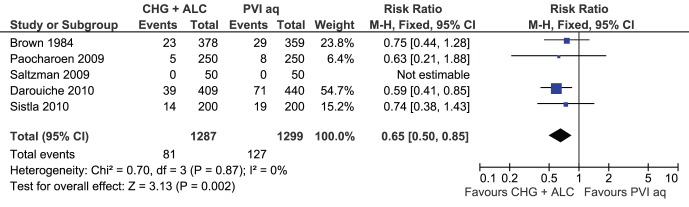
Meta-analysis of skin antiseptics for the prevention of surgical site infection. CHG plus alcohol versus aqueous PVI. References and abbreviations are as provided in Table 3.

Five RCTs evaluated CHG-alcohol versus aqueous PVI and were included in a meta-analysis (Figure 4). This showed a significant advantage of CHG-alcohol in reducing surgical site infections (RR 0.65; 95% CI: 0.50–0.85). The remaining RCTs evaluated various CHG-alcohol against various iodine-alcohol combinations, but these studies were very heterogeneous. For two larger trials [50], [53], the types and concentrations of alcohol could not be clarified. Two smaller trials [52], [56] had satisfactory alcohol concentrations but had few outcomes only, and one trial [54] used an alcohol concentration (23%) far below the antimicrobially active range in the PVI-containing arm. Given that different alcohol types and concentrations can easily tip the efficacy in favour of one or another preparation [61], we elected not to perform additional meta-analyses. Again, the non-RCT studies are listed in Table 3 but were not included in meta-analyses.

The first systematic review [60] included 7 trials, of which only one had a CHG-containing arm. It concluded that there was insufficient evidence to support a particular antiseptic over another. Another systematic review [12] included 9 trials comparing CHG-containing versus iodine-containing antiseptics. The authors examined two outcomes, surgical site infections and microbial skin cultures. The majority of studies (5 trials) compared CHG-alcohol with aqueous PVI. The authors pooled all CHG-containing versus all iodine-containing trials – without accounting for other ingredients – and found a significant risk reduction for both outcomes in favour of the CHG-containing preparations. The conclusion was that skin antisepsis with CHG is more effective than with iodine. We further examined the included articles that assessed microbial skin cultures and found that none reported whether neutralisers were used in the testing. However, suitable neutralisers are essential for antimicrobial efficacy assessment [62], [63]. The third systematic review [13] examined six trials; the authors pooled any CHG-containing versus any PVI-containing antiseptics without accounting for other ingredients and concluded that CHG *per se* was superior to povidone-iodine *per se*.

Again, we found examples of tertiary publications that contained unsubstantiated statements about the role of CHG. A narrative Current Concepts review on the prevention of perioperative infection [64] concluded: “chlorhexidine gluconate is superior to povidone-iodine for preoperative antisepsis”. A surgical care initiative by Washington State hospitals [65] announced that it would mandate that skin preparation should be done with “chlorhexidine”, citing the study of Darouiche et al [6]. The 2010 national Australian infection control guidelines [10] state that “chlorhexidine” (without reference to alcohol) should preferably be used for skin preparation in surgery. The UK National Institute for Health and Clinical Excellence (NICE) issued a public review proposal for its surgical guidelines [66], citing new evidence of benefits of CHG over PVI for surgical skin preparation.

As for blood cultures, we did not find any relevant evidence supporting CHG alone for pre-incisional preparation of superficial skin in surgery. In fact, aqueous CHG commonly fails US regulatory requirements for patient preoperative skin preparation [67], [68].

## Discussion

We found a high proportion of primary and secondary literature and some prominent tertiary sources that attributed the efficacy of the combination of CHG and alcohol to CHG alone. The rates of incorrect attribution among the articles that we assessed ranged from 29% for catheters to 43% for surgery. The rates of incorrect and ambiguous attribution combined ranged from 42% for blood cultures to 65% for catheters. For surgery, there were more articles with incorrect (43%) than with correct attribution (36%). These conclusions were found at all levels of evidence gathering and knowledge translation, including primary clinical trials, systematic reviews, clinical practice recommendations and evidence-based guidelines.

The omission of alcohols in the process of evidence assessment can be seen, for example, in the draft CDC catheter guidelines [9] which initially recommended CHG alone for central venous catheter insertion and maintenance. This was subsequently changed to CHG-alcohol in the final guidelines [49]. We are unaware of the sequence of events, but assume that the change may have come through external submissions during the public comment phase. This change effectively rectified the section on skin antisepsis in the final guidelines. Another area of impact is a potentially mistaken rejection of alternatives or competitor products on the basis that they do not contain CHG, even if they have not been sufficiently tested in clinical trials. This appears to be affecting PVI plus alcohol in surgery, by way of negative implication [10], [12], [13], [65], [66].

In our analyses, we found good evidence favouring CHG-alcohol combinations over aqueous PVI, the most commonly tested alternative, in all three areas of skin antisepsis. This is a comparison of two active agents against one. However, this superiority does not hold against PVI plus alcohol or other competitors combined with alcohol, either due to equivalent performance in meta-analyses (for blood cultures) or a lack of relevant studies (for catheters and surgery). For blood cultures, alcohols alone may be effective, according to another analysis [26]. For surgery, the question of CHG-alcohol versus iodine-alcohol is unresolved. For both blood cultures and surgery, we found no evidence that CHG alone is effective. For vascular catheters, the situation is more complex. There is evidence that CHG alone is superior to PVI alone for preventing colonisation, but its effect did not reach significance for CR-BSI. In contrast, CHG-alcohol was superior to PVI alone for both outcomes, colonisation and CR-BSI.

Each of the three applications has different biological and functional requirements. Blood culture collection requires immediate activity at the venipuncture site, but no prolonged action. Alcohols, with their strong immediate activity that typically exceeds those of CHG and PVI by about a factor of 10 [1], [2], [61], fulfill this requirement well. Surgery requires significant immediate activity before incision and some persistent activity during the operation for several hours. Thus, surgical skin preparation is expected to benefit from the immediate action of alcohols plus persistent or enhanced activity from added CHG or PVI [67], [69]. Vascular catheter sites also require good immediate activity before insertion, but since catheters often stay in place for a week or more, good persistent action is also required. This requirement is fulfilled well by CHG [1], [2], [67].

Another fact deserves consideration. Most catheter studies used the antiseptics both before insertion and during maintenance (Table 2). Thus, when viewed strictly, it is not known exactly from the study results whether the antiseptics were more effective at the point of insertion or during maintenance, and which component would be better suited to which phase.

Our review has several limitations. First, it is partially hypothesis-driven, as indicated at the end of the Introduction section. This is unusual for systematic reviews, but nevertheless we used an explicit and rigorous systematic review methodology, including adherence to the PRISMA Statement [70]. Second, our assessment of authors’ attribution is partially based on subjective judgement. This judgement was straightforward in virtually all articles classified as “incorrect”. We tried to err on the side of caution and assigned all articles in which this was less clear to the “intermediate” category. Third, since our search and assessment strategy focussed on any chlorhexidine-containing versus any other antiseptics, our review is not comprehensive in terms of capturing relevant comparisons between different non-CHG-based antiseptics in the three areas. Fourth, we faced limitations from heterogeneity in study design, differences in antiseptic compositions, and a lack of other relevant information while performing our analyses. Attribution was assessed for all RCTs, non-RCTs and systematic reviews, because this was not affected by study design. However, only RCTs were included in meta-analyses, in which only studies with the same basic antiseptic components were pooled together. Nevertheless, due to inherent differences between antiseptic products, we had to retain some variability of antiseptic concentrations and alcohol types in our analyses.

We were unable to trace the exact origins of the CHG misattribution. However – even though this is speculative – some observations in the literature provide a few potential reasons. First, some authors may regard alcohol simply as a solvent for CHG. This is reflected by the commonly-used term “chlorhexidine in alcohol” and references to alcohol as a “base solution” for CHG. Second, alcohol may not be universally regarded as an effective antiseptic. For example, wording in the CLSI blood culture guideline [7] suggests that it is viewed as a cleansing agent at the venipuncture site. Third, some authors may be using the term “chlorhexidine” to actually mean the CHG-alcohol combination. This is suggested by some text passages in the draft CDC catheter guidelines [9]. In any case, this would constitute incorrect usage of the term.

Our findings have broader implications. An important scientific principle – the fact that it is generally not possible to attribute effects to only one factor when several factors have been tested together – has frequently been overlooked. The individual published analyses may have been done correctly at a technical level of evidence assessment [14], but the conclusions appear incorrect. What are possible causes, and what are further implications?

First, it may be a matter of subjective views held by authors. If, for example, alcohol is regarded as a mere solvent for CHG, then authors are unable to draw appropriate conclusions. This means that the assessment of evidence remains susceptible to subjective influences, and this will continue to require attention in this area as well as in other subject areas. Second, this highlights the principle of biological plausibility. In the CHG example, plausibility could have been checked by what is known from microbiological studies of antiseptics [1], [2], [61], [67], [69]; this would have indicated that alcohol is a key component. While biological plausibility is part of the Bradford-Hill Criteria in epidemiological studies [71], there is currently no explicit requirement to address this in clinical trials and systematic reviews [14], [72]. However, we think this should become a requirement.

Our findings also have potential implications for patient safety. When following recommendations to use “chlorhexidine”, caregivers may inappropriately use CHG on its own, in aqueous solution, as this is readily available. The clinical impact from blood cultures and vascular catheterisation may be small, because contaminated blood cultures do not directly harm patients and CHG alone appears to exert some protective effect in vascular catheterisation. However, tangible negative consequences may arise in surgery, because marked differences in surgical infection rates have been observed between different antiseptic regimens [6], [57]. Conversely, if caregivers are unaware of the presence and significance of alcohols, they might accidentally use alcohol compounds for antisepsis on mucous membranes, where they are contraindicated.

In summary, there is good evidence that CHG-alcohol is superior to aqueous PVI – an important competitor – in all three areas of skin antisepsis. However, this does not apply to competitors combined with alcohols. The perceived efficacy of CHG in skin antisepsis is often in fact based on evidence for the efficacy of the CHG-alcohol combination. In conjunction, the role of alcohol has frequently been overlooked in evidence assessments. This has broader implications for knowledge translation as well as potential implications for patient safety.

## Supporting Information

Table S1 **Results of risk of bias assessment for studies evaluating antiseptics for blood culture collection.**
(PDF)

Table S2 **Results of risk of bias assessment for studies evaluating antiseptics for vascular catheter insertion.**
(PDF)

Table S3 **Results of risk of bias assessment for studies evaluating antiseptics for surgical skin preparation.**
(PDF)

Text S1 **Eligibility criteria, literature search strategy and risk of bias assessment.**
(PDF)

Checklist S1 **PRISMA Checklist.**
(PDF)
