# Supplementary material for: The Forgotten Role of Alcohol: A Systematic Review and Meta-Analysis of the Clinical Efficacy and Perceived Role of Chlorhexidine in Skin Antisepsis
Source: PLoS One. 2012 Sep 5;7(9):e44277. doi: 10.1371/journal.pone.0044277 (PMC3434203; doi:10.1371/journal.pone.0044277)
Supplement: Table S1 — Results of risk of bias assessment for studies evaluating antiseptics for blood culture collection. (PDF) [file pone.0044277.s001.pdf]

Table S1. Results of risk of bias assessment for studies evaluating antiseptics for blood culture collection.

Study	Randomisation	Allocation conceal-ment	Blinding of inter-vention	Blinding of outcome assess-ment	Incom-plete outcome data	Selective reporting	Study groups equal at baseline	Other bias	Ratings summary	Overall risk of bias
Mimoz et al. 1999 [16]	A	B	C	A	B	A	A	A	5 A, 2 B, 1 C	Low
Trautner et al. 2002 [17]	A	B	C	A	A	A	A	A	6 A, 1 B, 1 C	Low
Suwanpimolkul et al. 2008 [22]	B	B	C	A	B	A	B	C	2 A, 4 B, 2 C	Moderate
Washer et al. 2010 [24]	B	C	C	A	B	A	B	C	2 A, 3 B, 3 C	High

Notes. Bias rankings: A, low risk of bias; B, uncertain risk of bias; C, high risk of bias. The overall risk of bias was judged by the authors, taking into account the relative importance of the criteria.
