# Supplementary material for: The Forgotten Role of Alcohol: A Systematic Review and Meta-Analysis of the Clinical Efficacy and Perceived Role of Chlorhexidine in Skin Antisepsis
Source: PLoS One. 2012 Sep 5;7(9):e44277. doi: 10.1371/journal.pone.0044277 (PMC3434203; doi:10.1371/journal.pone.0044277)
Supplement: Table S2 — Results of risk of bias assessment for studies evaluating antiseptics for vascular catheter insertion. (PDF) [file pone.0044277.s002.pdf]

Table S2. Results of risk of bias assessment for studies evaluating antiseptics for vascular catheter insertion.

Study	Randomisation	Allocation conceal-ment	Blinding of inter-vention	Blinding of outcome assess-ment	Incom-plete outcome data	Selective reporting	Study groups equal at baseline	Other bias	Ratings summary	Overall risk of bias
Maki et al. 1991 [28]	B	B	C	A	B	A	C	C	2 A, 3 B, 3 C	Moderate
Sheehan et al. 1993 [29]	B	B	C	B	A	A	A	B	3 A, 4 B, 1 C	Moderate
Meffre et al. 1996 [31]	B	B	C	B	A	A	C	B	2 A, 4 B, 2 C	Moderate
Mimoz et al. 1996 [32]	A	A	C	A	B	A	C	A	5 A, 1 B, 2 C	Low
Legras et al. 1997 [33]	B	B	C	B	A	A	A	A	4 A, 3 B, 1 C	Low
Cobbett and LeBlanc 2000 [34]	B	B	C	B	B	C	A	A	2 A, 4 B, 2 C	Moderate
Humar et al. 2000 [35]	A	A	C	A	A	A	A	A	7 A, 0 B, 1 C	Low
Maki et al. 2001 [36]	B	B	C	A	A	A	B	B	3 A, 4 B, 1 C	Moderate
Langgartner et al. 2004 [37]	B	A	C	B	B	B	A	A	3 A, 4 B, 1 C	Moderate
Astle and Jensen 2005 [38]	B	B	C	B	B	C	A	A	2 A, 4 B, 2 C	Moderate
Kelly et al. 2005 [39]	B	B	C	B	B	A	A	B	2 A, 5 B, 1 C	Moderate
Mimoz et al. 2007 [41]	A	A	C	A	A	A	B	A	6 A, 1 B, 1 C	Low
Small et al. 2008 [42]	B	B	C	B	B	A	B	C	1 A, 5 B, 2 C	High
Vallés et al. 2008 [43]	B	A	C	A	B	A	C	C	3 A, 2 B, 3 C	Moderate
Garland et al. 2009 [44]	B	B	C	B	A	A	A	A	4 A, 3 B, 1 C	Low

Notes. Bias rankings: A, low risk of bias; B, uncertain risk of bias; C, high risk of bias. The overall risk of bias was judged by the authors, taking into account the relative importance of the criteria.
