# Supplementary material for: The Forgotten Role of Alcohol: A Systematic Review and Meta-Analysis of the Clinical Efficacy and Perceived Role of Chlorhexidine in Skin Antisepsis
Source: PLoS One. 2012 Sep 5;7(9):e44277. doi: 10.1371/journal.pone.0044277 (PMC3434203; doi:10.1371/journal.pone.0044277)
Supplement: Table S3 — Results of risk of bias assessment for studies evaluating antiseptics for surgical skin preparation. (PDF) [file pone.0044277.s003.pdf]

Table S3. Results of risk of bias assessment for studies evaluating antiseptics for surgical skin preparation.

Study	Randomisation	Allocation concealment	Blinding of intervention	Blinding of outcome assessment	Incomplete outcome data	Selective reporting	Study groups equal at baseline	Other bias	Ratings summary	Overall risk of bias
Berry et al. 1982 [50]	A	A	C	A	A	A	A	C	6 A, 0 B, 2 C	Low
Brown et al. 1984 [51]	B	A	C	C	A	B	C	A	3 A, 2 B, 3 C	Moderate
Ostrander et al. 2005 [52]	C	B	C	B	A	B	B	B	1 A, 5 B, 2 C	High
Veiga et al. 2008 [53]	B	B	C	B	A	A	B	A	3 A, 4 B, 1 C	Moderate
Cheng et al. 2009 [54]	A	B	C	B	A	B	A	A	4 A, 3 B, 1 C	Low
Paocharoen et al. 2009 [55]	B	B	C	B	A	A	A	A	4 A, 3 B, 1 C	Low
Saltzman et al. 2009 [56]	B	A	C	B	A	A	C	A	4 A, 2 B, 2 C	Moderate
Darouiche et al. 2010 [6]	A	B	C	A	A	A	A	A	6 A, 1 B, 1 C	Low
Sistla et al. 2010 [58]	B	A	C	A	B	A	A	B	4 A, 3 B, 1 C	Low

Notes. Bias rankings: A, low risk of bias; B, uncertain risk of bias; C, high risk of bias. The overall risk of bias was judged by the authors, taking into account the relative importance of the criteria.
