# Supplementary material for: The Forgotten Role of Alcohol: A Systematic Review and Meta-Analysis of the Clinical Efficacy and Perceived Role of Chlorhexidine in Skin Antisepsis
Source: PLoS One. 2012 Sep 5;7(9):e44277. doi: 10.1371/journal.pone.0044277 (PMC3434203; doi:10.1371/journal.pone.0044277)
Supplement: Text S1 — Eligibility criteria, literature search strategy and risk of bias assessment. (PDF) [file pone.0044277.s004.pdf]

Supplementary Text S1

The forgotten role of alcohol: a systematic review and meta-analysis of the clinical efficacy and perceived role of chlorhexidine in skin antisepsis

Matthias Maiwald, Edwin S.-Y. Chan

Eligibility Criteria for Blood Culture Articles

Participants: Human patients in clinical settings undergoing skin antisepsis prior to venipuncture for blood culture collection. **Comparisons:** Any chlorhexidine-containing skin antiseptic versus any other comparator antiseptic. **Outcomes:** Blood culture contamination (binary). Attribution criterion: Did the authors examine the combination of chlorhexidine and alcohol and attribute any outcomes from this combination solely to chlorhexidine? **Study design:** Primary clinical studies (both randomised clinical trials and non-randomised clinical studies) and systematic reviews. Only randomised clinical trials were considered for meta-analyses, but all primary literature and systematic reviews were to be assessed by the attribution criterion. **Exclusion criteria:** Articles where skin antisepsis was studied only as one part of a multifactorial intervention together with other factors.

Eligibility Criteria for Vascular Catheter Articles

Participants: Human patients in clinical settings undergoing skin antisepsis prior to insertion, or both prior to insertion and during maintenance, of vascular catheters, including central venous, arterial, peripherally inserted central venous, and peripheral venous catheters. **Comparisons:** Any chlorhexidine-containing skin antiseptic versus any other comparator antiseptic. **Outcomes:** Microbial catheter colonisation or catheter-related bloodstream infections, or both (binary). Attribution criterion: Did the authors examine the combination of chlorhexidine and alcohol and attribute any outcomes from this combination solely to chlorhexidine? **Study design:** Primary clinical studies (both randomised clinical trials and non-randomised clinical studies) and systematic reviews. Only randomised clinical trials were considered for meta-analyses, but all primary literature and systematic reviews were to be assessed by the attribution criterion. **Exclusion criteria:** Articles where skin antisepsis was studied only as one part of a multifactorial intervention together with other factors. Articles where antisepsis was studied only during maintenance, or only performed on catheter hubs or connectors.

Eligibility Criteria for Surgery Articles

Participants: Human patients in clinical settings undergoing skin antisepsis on superficial skin in the operating room setting prior to surgery (classical surgical skin preparation), including any type of surgery through superficial skin. **Comparisons:** Any chlorhexidine-containing skin antiseptic versus any other comparator antiseptic. **Outcomes:** Surgical site infection (binary). Attribution criterion: Did the authors examine the combination of chlorhexidine and alcohol and attribute any outcomes from this combination solely to chlorhexidine? **Study design:** Primary clinical studies (both randomised clinical trials and non-randomised

clinical studies) and systematic reviews. Only randomised clinical trials were considered for meta-analyses, but all primary literature and systematic reviews were to be assessed by the attribution criterion. **Exclusion criteria:** Articles where skin antisepsis was studied only as one part of a multifactorial intervention together with other factors. Articles studying antiseptic cloth wiping or antiseptic bathing or showering in the phase leading up to an operation (e.g. at home or on the evening before an operation), or antisepsis for mucous membrane surgery.

Literature search dates and limits

Literature searches were performed during August to October 2011. No specific limits (dates of coverage, language) were applied.

Search Strategy for Blood Culture Articles

PubMed initial search (search terms “chlorhex* AND blood culture”) – 72 records
PubMed alternative search:
#1: chlorhex* AND blood culture
#2: "Chlorhexidine"[Majr]
#3: "chlorhexidine gluconate"[supplementary concept]
#4: chlorhex*
#5: ((#2) OR #3) OR #4
#6: blood/microbiology*
#7: Blood Specimen Collection/methods*
#8: "blood culture"
#9: blood culture
#10: (((#6) OR #7) OR #8) OR #9
#11: (#5) AND #10 – 76 records
#12: (#11) NOT #1 – 4 records

CINAHL on EBSCOhost (search terms “chlorhex* AND blood AND culture”) – 30 records
Cochrane Library (search terms “chlorhex* AND blood culture”) – 35 records
Agency for Healthcare Research and Quality website – 65 records
ClinicalTrials.gov website (http://clinicaltrials.gov) – 268 records
WHO Clinical Trials registry (http://apps.who.int/trialsearch) – 122 records
Current Controlled Trials website (http://www.controlled-trials.com) – 153 records
If no specific search terms are given, the single term “chlorhexidine” was used.
A chlorhexidine product website, UK section (CareFusion, San Diego, CA, USA), manual search (http://www.chloraprep.co.uk/publishedStudies.html) – 37 records

Records were scanned for articles that might meet the inclusion criteria. Two systematic reviews were found [25,26]; their reference sections were scanned for additional articles (total 41 references). A total of 16 records

were retrieved in full, of which 4 articles were excluded after review. Reasons for exclusion were: did not evaluate blood culture contamination (n = 1), trial database entry, no publishable results yet (n = 1), multifactorial intervention, not just different antiseptics (n = 2).

A total of 12 articles were included in the qualitative synthesis (all articles in Table 1) and 4 articles were included in the quantitative synthesis (randomised trials in Table 1). A flow diagram is shown in Figure 1.

Search Strategy for Vascular Catheter Articles

PubMed initial search (search terms “chlorhex* AND catheter*”) – 472 records
PubMed alternative search:
#1: chlorhex* AND catheter*
#2: "Chlorhexidine"[Majr]
#3: "chlorhexidine gluconate"[supplementary concept]
#4: chlorhex*
#5: ((#2) OR #3) OR #4
#6: "catheters"[Mesh]
#7: catheter*
#8: (#6) OR #7
#9: (#5) AND #8 – 472 records
#10: (#9) NOT #1 – 0 records

CINAHL on EBSCOhost (search terms “chlorhex* AND catheter*”) – 244 records
Cochrane Library (search terms “chlorhex* AND catheter*”) – 127 records
Agency for Healthcare Research and Quality website – 65 records
ClinicalTrials.gov website (http://clinicaltrials.gov) – 268 records
WHO Clinical Trials registry (http://apps.who.int/trialsearch) – 122 records
Current Controlled Trials website (http://www.controlled-trials.com) – 153 records
If no specific search terms are given, the single term “chlorhexidine” was used.
A chlorhexidine product website, UK section (CareFusion, San Diego, CA, USA), manual search (http://www.chloraprep.co.uk/publishedStudies.html) – 37 records

Records were scanned for articles that might meet the inclusion criteria. Two systematic reviews were found [46,47]; their reference sections were scanned for additional articles (total 97 references). One of these systematic reviews [47] was not available in public databases. It was located on the Australian National Health and Medical Research Council (NHMRC) website after it was publicly announced and released with the 2010 Australian Infection Control Guidelines. A total of 50 records were retrieved in full, of which 30 articles were excluded after review. Reasons for exclusion were: comparator arm used no antiseptic (n = 1), antiseptic only studied in the form of insertion site dressing (n = 7), only catheter maintenance, not insertion studied (n = 3), peritoneal, not vascular catheters studied (n =1), antiseptics used for cannulation, not catheter insertion (n = 1),

does not satisfy criteria for systematic review or is narrative review (n = 3), neither catheter colonisation nor catheter-related bloodstream infection reported (n = 2), entry in trial database, no published results, attempt to contact author unsuccessful (n = 1), only mathematical decision analysis, no actual study (n = 1), multifactorial intervention (n = 1), antiseptics tested only for line access (n = 3), unclear what exactly was studied in abstract, attempt to contact author unsuccessful (n = 1), trial of antibiotic-impregnated catheters, not skin antiseptics (n = 1), chlorhexidine used for body washes, not insertion site antisepsis (n = 1), redundant publication of the same studies (n = 3).

A total of 20 articles were included in the qualitative synthesis (all articles in Table 2) and 15 articles were included in the quantitative synthesis (randomised trials in Table 2). A flow diagram is shown in Figure 1.

Search Strategy for Surgery Articles

PubMed initial search (search terms “chlorhex* AND (surg* OR surgery OR surgical)”) – 1621 records
PubMed alternative search:
#1: chlorhex* AND (surg* OR surgery OR surgical)
#2: "Chlorhexidine"[Majr]
#3: "chlorhexidine gluconate"[supplementary concept]
#4: chlorhex*
#5: ((#2) OR #3) OR #4
#6: "General Surgery"[Majr]
#7: "Surgical Procedures, Operative"[Majr]
#8: surgery
#9: surgical
#10: Search (((#6) OR #7) OR #8) OR #9
#11: Search (#5) AND #10 – 1608 records
#12: Search (#10) NOT #1 – 0 records

CINAHL on EBSCOhost (search terms “chlorhex* AND surg*”) – 341 records
Cochrane Library (search terms “chlorhex* AND surg*”) – 328 records
Agency for Healthcare Research and Quality website – 65 records
ClinicalTrials.gov website (http://clinicaltrials.gov) – 268 records
WHO Clinical Trials registry (http://apps.who.int/trialsearch) – 122 records
Current Controlled Trials website (http://www.controlled-trials.com) – 153 records
If no specific search terms are given, the single term “chlorhexidine” was used.
A chlorhexidine product website, UK section (CareFusion, San Diego, CA, USA), manual search (http://www.chloraprep.co.uk/publishedStudies.html) – 37 records

Records were scanned for articles that might meet the inclusion criteria. Three systematic reviews were found [12,13,60]; their reference sections were scanned for additional articles (total 136 references). A total of 49

records were retrieved in full, of which 35 articles were excluded after review. Reasons for exclusion were: no involvement of chlorhexidine as antiseptic (n = 1), chlorhexidine used for urinary bladder antisepsis (n = 1), only microbial skin counts, no surgical site infections assessed (n = 7), healthy volunteers studied in terms of skin counts (n = 5), antiseptic body washing, not skin preparation (n = 5), surgical hand antisepsis, not skin antisepsis studied (n = 3), narrative, not systematic review (n = 3), same antiseptics used in both study arms (n = 4), mucous membrane, not superficial skin antisepsis (n = 1), comparator arm used no antiseptic (n = 1), entry in trial database, no published results, attempt to contact author unsuccessful (n = 3), entry in trial database, author contacted, nothing to report yet (n = 1).

A total of 14 articles were included in the qualitative synthesis (all articles in Table 3) and 9 articles were included in the quantitative synthesis (randomised trials in Table 3). A flow diagram is shown in Figure 1.

Risk of Bias in Individual Studies

Risk of bias in individual studies was assessed using the tools available in the RevMan Version 5.1 software by the Cochrane Collaboration (http://ims.cochrane.org/RevMan) and rated according to the criteria specified in the Cochrane Handbook [14] and the PRISMA Statement [70]. The final set of criteria consisted of: (i) random sequence generation (selection bias), (ii) allocation concealment (selection bias), (iii) blinding of participants and personnel (performance bias), (iv) blinding of outcome assessment (detection bias), (v) incomplete outcome data (attrition bias), (vi) selective reporting (reporting bias), (vii) study groups equal at baseline, and (viii) any other bias. The data were entered into RevMan and transferred into risk of bias tables (Supplementary Tables S1-S3). Earlier types of numerical scales for risk of bias assessment were not pursued, consistent with current recommendations by the Cochrane Collaboration [14].

Overall, the analysis revealed a mixed picture between different studies, but a few distinct characteristics were noted. None of the studies was able to blind patients and caregivers, due to the obvious appearance differences between the antiseptics. Many studies did not report on the method of random sequence generation and whether the study group allocation was adequately concealed. Adequate blinding of outcome assessment was more commonly but not universally reported. Incomplete outcome data (attrition) again constituted a mixed picture. Potential selective reporting was uncommon. The majority of studies in the three areas of skin antisepsis had study groups that were equal at baseline; some did not provide sufficient information, and only five studies had groups that had mostly only a few statistically significant differences between them.

A total of six studies received “high” ratings in the “other bias” category. Among the blood cultures, the trial of Suwanpimolkul et al [22] was generally well conducted, but had a minor part where patients had not been truly randomised, and the trial of Washer et al [24] was a cluster-randomised trial with wards as the unit of randomisation. Among the catheter studies, the trials of Maki et al [28], Small et al [42] and Vallés et al [43] received “high” ratings because of inconsistencies in data interpretation and reporting. Among the surgical studies, the study of Berry et al [50] had a likely source of other bias in that the observation time of 3-4 days for assessing surgical site infections was far too short for a valid assessment.

Among the ways of dealing with risk of bias within studies, we decided not to exclude any of the studies with a summary “high” rating from the final analyses. Instead, we evaluated an alternative scenario of excluding the ones with a “high” rating from the meta-analyses, and this did not result in a change of the overall results concerning the clinical efficacy of chlorhexidine versus other antiseptics; it also did not affect our overall conclusions (data not shown).

Risk of Bias Across Studies

Risk of bias across studies was difficult to evaluate due to the small numbers of studies in each comparison. The number of studies in our meta-analyses ranged between two (for blood) and eight (for catheters). The use of statistical methods to assess bias across studies is discouraged when that number is below ten, and there are some inherent difficulties in interpreting funnel plots as a measure of publication bias [14,73].

For each of the meta-analyses, statistical heterogeneity was calculated and funnel plots were generated within RevMan. Funnel plots were visually inspected for signs of asymmetry, and potential sources investigated. Similarly, potential sources were investigated in the presence of statistical heterogeneity. For the two blood culture meta-analyses, there were only two studies in each, thus precluding meaningful analysis. For the catheter studies, the comparison of aqueous chlorhexidine versus aqueous povidone-iodine in terms of catheter colonisation (Figure 3A) revealed statistical heterogeneity. The study by Vallés et al [43] was identified as the source of this heterogeneity; this study was similar in size but had a greater incidence of catheter colonisation than the two other trials. For the comparison of chlorhexidine-alcohol versus aqueous povidone-iodine in terms of catheter colonisation (Figure 3C), there was a relatively flat funnel plot with most studies having similar sizes, and statistical heterogeneity was detected. Sources of this heterogeneity were the trials of Humar et al [35] and Maki et al [36], which were both large trials with outcomes located at opposite ends of the measured effects spectrum. Alternative examination by exclusion of both trials did not change the significance and overall effect size of this comparison (data not shown). Alternative examination using a fixed-effects versus a random-effects model for calculation also did not change the significance for the datasets in Figure 3A and 3C. For the comparison of chlorhexidine-alcohol versus aqueous povidone-iodine in terms of catheter-related bloodstream infection (Figure 3D), there appeared to be funnel plot asymmetry in the form of two smaller studies favouring chlorhexidine-alcohol [33,39]. Alternative examination by exclusion of these studies did not change significance for this outcome. For the surgical studies, the comparison of chlorhexidine-alcohol versus aqueous povidone-iodine (Figure 4) showed a symmetrical funnel plot and no statistical heterogeneity.

References

References [1] to [72] are provided in the main article. Additional reference in the supplement:

73. Sterne JA, Sutton AJ, Ioannidis JP, Terrin N, Jones DR, et al. (2011) Recommendations for examining and interpreting funnel plot asymmetry in meta-analyses of randomised controlled trials. BMJ 343: d4002.
