# Supplementary material for: The Forgotten Role of Alcohol: A Systematic Review and Meta-Analysis of the Clinical Efficacy and Perceived Role of Chlorhexidine in Skin Antisepsis
Source: PLoS One. 2012 Sep 5;7(9):e44277. doi: 10.1371/journal.pone.0044277 (PMC3434203; doi:10.1371/journal.pone.0044277)
Supplement: Checklist S1 — PRISMA Checklist. (PDF) [file pone.0044277.s005.pdf]

PRISMA 2009 Checklist

M. Maiwald, E. S.-Y. Chan. The forgotten role of alcohol: a systematic review and meta-analysis of the clinical efficacy and perceived role of chlorhexidine in skin antisepsis.

Section/topic	#	Checklist item	Reported on page #
**TITLE**			
Title	1	Identify the report as a systematic review, meta-analysis, or both.	Title page
**ABSTRACT**			
Structured summary	2	Provide a structured summary including, as applicable: background; objectives; data sources; study eligibility criteria, participants, and interventions; study appraisal and synthesis methods; results; limitations; conclusions and implications of key findings; systematic review registration number.	Abstract page
**INTRODUCTION**			
Rationale	3	Describe the rationale for the review in the context of what is already known.	Introduction, 4th and 5th paragraph
Objectives	4	Provide an explicit statement of questions being addressed with reference to participants, interventions, comparisons, outcomes, and study design (PICOS).	Introduction, 5th paragraph, and supplement*
**METHODS**			
Protocol and registration	5	Indicate if a review protocol exists, if and where it can be accessed (e.g., Web address), and, if available, provide registration information including registration number.	N/A†
Eligibility criteria	6	Specify study characteristics (e.g., PICOS, length of follow-up) and report characteristics (e.g., years considered, language, publication status) used as criteria for eligibility, giving rationale.	Methods section, 2nd paragraph and supplement
Information sources	7	Describe all information sources (e.g., databases with dates of coverage, contact with study authors to identify additional studies) in the search and date last searched.	Methods section, 1st paragraph and supplement
Search	8	Present full electronic search strategy for at least one database, including any limits used, such that it could be repeated.	Supplement text S1
Study selection	9	State the process for selecting studies (i.e., screening, eligibility, included in systematic review, and, if applicable, included in the meta-analysis).	Methods section, 2nd and 3rd paragraph; Figure 1; supplement text
Data collection process	10	Describe method of data extraction from reports (e.g., piloted forms, independently, in duplicate) and any processes for obtaining and confirming data from investigators.	Methods section, 3rd paragraph
Data items	11	List and define all variables for which data were sought (e.g., PICOS, funding sources) and any assumptions and simplifications made.	Methods section, 3rd paragraph; supplement
Risk of bias in individual studies	12	Describe methods used for assessing risk of bias of individual studies (including specification of whether this was done at the study or outcome level), and how this information is to be used in any data synthesis.	Methods section, 3rd paragraph; supplement
Summary measures	13	State the principal summary measures (e.g., risk ratio, difference in means).	Methods section, 5th paragraph
Synthesis of results	14	Describe the methods of handling data and combining results of studies, if done, including measures of consistency (e.g., I^2) for each meta-analysis.	Methods section, 4th and 5th paragraph

PRISMA 2009 Checklist

Section/topic	#	Checklist item	Reported on page #
Risk of bias across studies	15	Specify any assessment of risk of bias that may affect the cumulative evidence (e.g., publication bias, selective reporting within studies).	Supplement
Additional analyses	16	Describe methods of additional analyses (e.g., sensitivity or subgroup analyses, meta-regression), if done, indicating which were pre-specified.	Methods section, 4th and 5th paragraph; supplement
**RESULTS**			
Study selection	17	Give numbers of studies screened, assessed for eligibility, and included in the review, with reasons for exclusions at each stage, ideally with a flow diagram.	Figure 1; supplement text
Study characteristics	18	For each study, present characteristics for which data were extracted (e.g., study size, PICOS, follow-up period) and provide the citations.	Tables 1-3
Risk of bias within studies	19	Present data on risk of bias of each study and, if available, any outcome level assessment (see item 12).	Supplement text; tables S1-S3
Results of individual studies	20	For all outcomes considered (benefits or harms), present, for each study: (a) simple summary data for each intervention group (b) effect estimates and confidence intervals, ideally with a forest plot.	Tables 1-3; Figures 2-4
Synthesis of results	21	Present results of each meta-analysis done, including confidence intervals and measures of consistency.	Results text; Figures 2-4
Risk of bias across studies	22	Present results of any assessment of risk of bias across studies (see Item 15).	Supplement
Additional analysis	23	Give results of additional analyses, if done (e.g., sensitivity or subgroup analyses, meta-regression [see Item 16]).	Supplement
**DISCUSSION**			
Summary of evidence	24	Summarize the main findings including the strength of evidence for each main outcome; consider their relevance to key groups (e.g., healthcare providers, users, and policy makers).	Discussion, 1st and 3rd paragraph
Limitations	25	Discuss limitations at study and outcome level (e.g., risk of bias), and at review-level (e.g., incomplete retrieval of identified research, reporting bias).	Discussion, 6th paragraph; supplement
Conclusions	26	Provide a general interpretation of the results in the context of other evidence, and implications for future research.	Discussion, last paragraph
**FUNDING**			
Funding	27	Describe sources of funding for the systematic review and other support (e.g., supply of data); role of funders for the systematic review.	Title page (no external support)

* Note: Not all items of the PRISMA Statement are covered in the main article. This was done to create an optimised flow of reading in the main article that focuses on the important major issues. All remaining information as formally required by PRISMA is provided in the supplement Text S1 and the Tables S1-S3.

† No prior review protocol exists, because this systematic review arose from an observation made in the literature (rationale described in the introduction).

From: Moher D, Liberati A, Tetzlaff J, Altman DG, The PRISMA Group (2009). Preferred Reporting Items for Systematic Reviews and Meta-Analyses: The PRISMA Statement. PLoS Med 6(6): e1000097. doi:10.1371/journal.pmed1000097
